# Antiproliferative and cytotoxic effects of sesquiterpene lactones isolated from *Ambrosia artemisiifolia* on human adenocarcinoma and normal cell lines

**DOI:** 10.1080/13880209.2022.2103574

**Published:** 2022-08-11

**Authors:** Balázs Kovács, Nikoletta Szemerédi, Norbert Kúsz, Tivadar Kiss, Boglárka Csupor-Löffler, Yu-Chi Tsai, Bálint Rácz, Gabriella Spengler, Dezső Csupor

**Affiliations:** a Institute of Pharmacognosy, Faculty of Pharmacy, University of Szeged, Szeged, Hungary; b Department of Medical Microbiology, Albert Szent-Györgyi Health Center, Albert Szent-Györgyi Medical School, University of Szeged, Szeged, Hungary; c Institute for Translational Medicine, Medical School, University of Pécs, Pécs, Hungary; d National Museum of Marine Biology and Aquarium, Pingtung, ROC; e Institute of Clinical Pharmacy, Faculty of Pharmacy, University of Szeged, Szeged, Hungary

**Keywords:** Ragweed, Asteraceae, 1,10-dihydro-1′-noraltamisin, human colonic

## Abstract

**Context:**

*Ambrosia artemisiifolia* L. (Asteraceae) contains sesquiterpene lactones as characteristic secondary metabolites. Many of these compounds exert antiproliferative and cytotoxic effects.

**Objective:**

To isolate the sesquiterpene lactones from the aerial part of *A. artemisiifolia* and to elucidate their cytotoxic, antiproliferative and antibacterial effects.

**Materials and methods:**

The compounds were identified by one-dimensional (1D) and 2D NMR, HR-MS spectroscopy from the methanol extract. Isolated compounds were investigated for their cytotoxic and antiproliferative effects on human colonic adenocarcinoma cell lines and human embryonal lung fibroblast cell line using MTT assay. The selectivity of the sesquiterpenes was calculated towards the normal cell line. To check the effect of drug interactions between compounds and doxorubicin, multidrug-resistant Colo 320 cells were used.

**Results:**

A new *seco*-psilostachyinolide derivative, 1,10-dihydro-1′-noraltamisin, and seven known compounds were isolated from the methanol extract. Acetoxydihydrodamsin had the most potent cytotoxic effect on sensitive (Colo205) cell line (IC_50_ = 7.64 µM), also the strongest antiproliferative effect on Colo205 (IC_50_ = 5.14 µM) and Colo320 (IC_50_ = 3.67 µM) cell lines. 1′-Noraltamisin (IC_50_ = 8.78 µM) and psilostachyin (IC_50_ = 5.29 µM) showed significant antiproliferative effects on the multidrug-resistant Colo320 cell line and had moderate selectivity against human embryonal lung fibroblast cell line. Psilostachyin C exhibited cytotoxic effects on Colo205 cells (IC_50_ = 26.60 µM). None of the isolated compounds inhibited ABCB1 efflux pump (EP; P-glycoprotein) or the bacterial EPs.

**Discussion and conclusions:**

Acetoxydihydrodamsin, 1′-noraltamisin, and psilostachyin showed the most remarkable cytotoxic and antiproliferative activity on tumour cell lines and exerted selectivity towards MRC-5 cell line.

## Introduction

Common ragweed (*Ambrosia artemisiifolia* L. [Asteraceae]) is an annual pioneer plant; the genus stems from the Sonoran Desert (USA) according to the phylogenetic studies (Payne 1964). Common ragweed is the most widespread species of the genus and its appearance has been recorded in central (Hungary, Austria and Slovakia), eastern (Ukraine), south-eastern (Croatia and Serbia) and southern (France and Italy) parts of Europe. Although, presently, *A. artemisiifolia* is relatively rare in the northern part of the continent (e.g., Ireland, Scotland, Norway and Sweden), the climate change and the great genetic variability of the plant can promote the infection of these regions in the near future (Hyvönen et al. 2011). As a weed, it produces large amount of highly allergenic pollen which can induce allergic disease, such as rhinitis, conjunctivitis and asthma. The two major allergens of ragweed pollen are the Amb a I and Amb a II endopeptidases with immunoglobulin-E binding capacity (King et al. 1964; Wopfner et al. 2009;). In addition, dermal exposure to the plant can cause contact dermatitis and urticaria, which has been described in other Asteraceae plants. This reaction can be attributed to the sesquiterpene lactones, representative compounds of this family (Möller et al. 2002). The widespread use of common ragweed in European folk medicine has not been documented, although ethnobotanical sources mention certain use of *A. artemisiifolia* by Native Americans for medicinal purposes (e.g., to treat insect bites, infected toes, minor skin eruptions, hives, tea used for fever and nausea). The medicinally used parts were the leaves and herbs. However, there are no detailed data on the developmental stages of the utilized plants (Speck 1941; Tantaquidgeon 1942; Romero 1954; Foster and Duke 1990).

Most of the *Ambrosia* sesquiterpenes possess of a C-11/C-13 exocyclic double bound conjugated to the *γ*-lactone which provides characteristic bioactivities to these secondary metabolites such as antiproliferative, cytotoxic and anti-inflammatory activities. These compounds alkylate nucleophiles, among them enzymes containing sulfhydryl groups which are crucial in cell proliferation and division and thus promote apoptosis in cancer cells (Kreuger et al. 2012). p65 is the specific target for sesquiterpenes, which is one of the members of the transcription factor NF-κB. This signalling pathway has a major role in tumour biology; it regulates key processes during initiation and progression of several types of cancer. In cancerous cells the NF-κB signalling promotes proliferation, angiogenesis, invasion, metastasis, chemoresistance and radio-resistance (Vazquez-Santillan et al. 2015). The tumour necrosis factor-α (TNF-α) is an activator of NF-κB. Sesquiterpene lactones damsin, ambrosin, coronopilin isolated from *A. arborescens* inhibited the TNF-α-induced translocation of NF-κB and all the compounds were cytotoxic to the breast cancer cell lines (MCF-7, JIMT-1 and HCC1937) (Sotillo et al. 2017). Compounds with other mechanisms of action, including G_2_ cell cycle checkpoint inhibitors (psilostachyin and psilostachyin C isolated from *A. artemisiifolia*) blocked MCF-7 mp53 cells in mitosis and caused the formation of aberrant microtubule spindles (Sturgeon et al. 2005). In cancer cells, the G_2_ checkpoint represents the cell’s defence against DNA damage. Psilostachyin and psilostachyin C as novel G_2_ checkpoint inhibitors offer a potential therapeutic approach to increase the efficacy of common DNA-damaging anticancer treatments.

Our study focussed on the isolation and identification of cytotoxic and antiproliferative secondary metabolites from common ragweed (Asteraceae), the characterization of their biological activities with special attention to the selective efficacy on cancer cell lines.

## Materials and methods

### General experimental procedure

Analytical-grade solvents were purchased from Chem-Lab NV (Zedelgem, Belgium). Solvents for extraction were obtained from Molar Chemicals Kft. (Halásztelek, Hungary). HPLC grade solvents were acquired from VWR Chemicals International S. A. S. (Fontenay-sous-Bois, France). HPLC grade water was purified by a Millipore Direct-Q^®^ 3 UV pump (Millipore S. A. S., Molsheim, France). Silica gel 60 RP-18 F_254_s and silica gel 60 F_254_ (Merck KGaA, Darmstadt, Germany) were used for TLC analysis. MN-Polyamide SC-6 (polycaprolactam, 0.05–0.16 mm, Macherey-Nagel GmbH & Co. KG, Düren, Germany) was applied for open column chromatography. The vacuum chromatography was carried out with a Büchi Rotavapor R-300 vacuum pump V-300 (BÜCHI Labortechnik AG, Flawil, Switzerland) using silica gel 60 GF_254_ (mean particle size 15 µm, Merck). The preparative rotation planar chromatography (RPC) was performed on a Harrison Chromatotron (Harrison Research, Palo Alto, CA), equipped with a Büchi pump Manager C-615 and with two Büchi Pump Module C-605 using silica gel (0.040–0.063 mm, Merck). Preparative HPLC was performed on a Shimadzu LC-2010 C_HT_ system (Shimadzu Corp., Tokyo, Japan), equipped with an UV–Vis detector with two channels, on-line degasser and autosampler using a Kinetex XB C_18_ (5 µm, 100 Å, 250 × 10.0 mm) column (Phenomenex, Torrance, CA). A Shimadzu LC-10AS system was applied, equipped with SPD-10A UV–Vis detector with single channel, on-line degasser and manual sampler using a Kinetex C_18_ (5 µm, 100 Å, 150 × 4.6 mm) column. NMR spectra were recorded in CDCl_3_ and CD_3_OD on a Bruker Avance DRX 500 spectrometer at 500 MHz (^1^H) and 125 MHz (^13^C JMOD). The residual peaks of the deuterated solvents were taken as reference points. Two-dimensional (2D) experiments were performed with a standard Bruker software. In the ^1^H–^1^H COSY, HSQC and HMBC experiments, gradient-enhanced version was applied. The data were acquired and processed with MesReNova version 11.0.4-18998 software (Mestrelab Research, Santiago de Compostela, Spain).

### Plant material

The raw plant material of *A. artemisiifolia* was collected in the flowering stage in July 2017 in the region of Mórahalom, Hungary. The above-ground parts were dried and stored at room temperature before processing. A voucher of specimen of the plant (No. 894) was deposited in the Herbarium of the Department of Pharmacognosy, University of Szeged.

### Extraction and isolation of sesquiterpenes from the above ground part

The dried plant material (5 kg) was ground with a Retsch^®^ SM 100 cutting mill and extracted with 51 L MeOH by ultrasonication at room temperature. The solvent was evaporated with a Büchi Rotavapor R-220 SE under reduced pressure. The extract was redissolved in 0.375 L H_2_O and 0.625 L MeOH and was subjected to solvent-solvent partitioning with chloroform (11 × 1 L). The weight of the crude chloroform fraction was 128.57 g. The CHCl_3_-partition fraction was chromatographed by open column chromatography on polyamide with a gradient system of MeOH-H_2_O with increasing MeOH concentration (from 20 to 100%) (17.5 L 20% MeOH, 30 L 40% MeOH, 42.5 L 60% MeOH and 55 L 80% MeOH), gaining 15 fractions (AA20/1, AA20/2, AA40/1, AA40/2, AA60/1-AA60/4, AA80/1-AA80/6 and AA100). Fraction AA20/1 (66 g) was subjected to vacuum chromatography on silica gel column with a gradient system of *n*-hexane-CHCl_3_-MeOH (50:30:00, 50:30:01, 50:30:02, 50:30:05, 50:30:10, 50:30:20, 50:30:30, 50:30:40, 50:30:50, 50:50:60, 50:60:70, 00:50:50, 100% MeOH) and gaining 10 fractions (AA/C1/C1-AA/C1/C10). Fraction AA/C1/C4 (3.83 g) eluted by *n*-hexane-CHCl_3_-MeOH (50:30:40) were further separated by RPC on silica gel and eluted with cyclohexane-EtOAc (16:4, 14:6, 12:8, 10:10, 8:12, 100% MeOH) with a flow rate 7 mL/min gaining 7 fractions (AA/C1/C4/R1*-AA/C1/C4/R7*). Fraction AA/C1/C4/R2* (433 mg) was purified by RPC on silica gel (gradient elution with cyclohexane-dichloromethane-MeOH 16:4:0.5, 14:5:0.5, 14:5:1, 12:7:1, 100% MeOH in the final step, flow rate 7 mL/min), 4 fractions were collected (AA/C1/C4/R2*/R1-AA/C1/C4/R2*/R4). Finally, compound **5** (17 mg) was obtained from fraction AA/C1/C4/R2*/R2 (184.9 mg) by using semipreparative HPLC gradient solvent system, consisting of methanol and water (0 min: MeOH-H_2_O 6.5:3.5, 6 min: MeOH:H_2_O 6.5:3.5, 9 min:MeOH:H_2_O 8:2, 10 min:MeOH:H_2_O 1:0, 11 min: MeOH:H_2_O 6.5:3.5, flow rate 2.8 mL/min)). Fraction AA/C1/C4/R6* (400 mg) was subjected to RPC using silica gel with gradient elution (cyclohexane-dichloromethane-MeOH 16:4:0.5, 14:5:0.5, 14:5:1, 12:7:1, 100% MeOH in the final step, flow rate 7 mL/min). Two fractions were collected AA/C1/C4/R6*/R1 (213.3 mg) and AA/C1/C4/R6*/R2 (138.4 mg). Fraction AA/C1/C4/R6^*/^R1 (213.3 mg) was dissolved in MeOH-dichloromethane 9:1, stored in the refrigerator for 24 h. Compound **2** (35 mg) was obtained by crystallization from this solvent mixture. Fraction AA/C1/C4/R5* (385 mg) was separated by RPC on silica gel with the same method gaining two fractions, AA/C1/C4/R5*/R1 (177.5 mg) and AA/C1/C4/R5*/R2 (161.1 mg). Fraction AA/C1/C4/R5*/R2 (161.1 mg) was further separated by RPC on silica gel eluting with toluene-acetone (18:2, 17:3, 16:4, 15:5, 100% MeOH in the final step), affording 6 fractions (AA/C1/C4/R5*/R2/R1-AA/C1/C4/R5*/R2/R6). Fraction AA/C1/C4/R5*/R2/R1 (10.5 mg) was purified by semipreparative HPLC (gradient elution, MeOH-H_2_O system, 0–1 min: MeOH-H_2_O 40:60, 1–12 min: MeOH-H_2_O 40 →100, 12–13 min: MeOH-H_2_O 100:0, 13–14 min: MeOH-H_2_O 100 → 40, flow rate 3.0 mL/min) to obtain compound **3** (6 mg). Fraction AA/C1/C4/R4* (380 mg) was subjected on silica gel and separated by RPC (gradient elution, cyclohexane-dichloromethane-MeOH 16:4:0.5, 14:5:0.5, 14:5:1, 12:7:1, 100% MeOH in the final step, flow rate 7 mL/min). Three fractions were collected (AA/C1/C4/R4*/R1-AA/C1/C4/R4*/R3). Fraction AA/C1/C4/R4*/R1 (104 mg) was dissolved in MeOH-dichloromethane 9:1, stored in the refrigerator for 24 h. Compound **6** (27 mg) was obtained by crystallization form this solvent system. Fraction AA/C1/C4/R4*/R2 (71.2 mg) was subjected to semipreparative HPLC (gradient solvent system, 0–1 min: MeOH-H_2_O 40:60, 1–10 min: MeOH 40 → 60, 10–12 min: MeOH-H_2_O 60:40, 12–15 min: MeOH 60 → 100, 15–16 min MeOH-H_2_O 100:0, 16–17 min: MeOH 100 → 40, flow rate 2.8 mL/min) to obtain compound **4** (34 mg). Fraction AA/C1/C4/R3* (415.2 mg) was purified by RPC using silica gel (gradient elution, cyclohexane-dichloromethane-MeOH 16:4:0.5, 14:5:0.5, 14:5:1, 12:7:1, 100% MeOH in the final step) and gaining 4 fractions (AA/C1/C4/R3*/R1-AA/C1/C4/R3*/R4). Fraction AA/C1/C4/R3*/R2 (149.9 mg) was further separated by RPC on silica gel (gradient elution, toluene-acetone 18:2, 17:3, 16:4, 15:5, 100% MeOH in the final step, flow rate 7 mL/min). Four fractions were collected (AA/C1/C4/R3*/R2/R1-AA/C1/C4/R3*/R2/R4). Subfraction AA/C1/C4/R3*/R2/R2 (30 mg) was purified by semipreparative HPLC (gradient solvent system, 0–1 min: MeOH–H_2_O 40:60, 1–10 min MeOH 40 → 60, 10–12 min: MeOH-H_2_O 64:40, 12–15 min: MeOH 60 → 100, 15–16 min: MeOH-H_2_O 100:0, 16–17 min: MeOH 100 → 40, flow rate 2.8 mL/min) to obtain compound **7** (4 mg). Fraction AA/C1C6 (4.12 g) was subjected to RPC with gradient elution system (cyclohexane-dichloromethane-MeOH 16:4:0.5, 14:5:0.5, 14:5:1, 12:7:1, 100% MeOH in the final step, flow rate 7 mL/min). Six fractions were collected (AA/C1/C6/R1*-AA/C1/C4/R6*). Fraction AA/C1/C6/R3* (126 mg) was further separated by RPC in the same way to gain five fractions (AA/C1/C6/R3*/R1-AA/C1/C6/R3*/R5). Subfraction AA/C1/C6/R3*/R1 (69 mg) was purified by HPLC (Shimadzu LC-10AS system, isocratic elution, MeOH-H_2_O 60:40, flow rate 0.8 mL/min) to obtain **1** (2 mg). Fraction AA/60/3 (3.4229 g) was chromatographed by open column chromatography on silica gel with a gradient system of *n*-hexane-EtOAc (9:1, 8:2, 4:1, 1:1, 1:4, 1:9 and 0:1) and EtOAc-MeOH (9:1, 4:1, 1:1, 1:4, 1:9 and 0:1) gaining 6 fractions (AA60/3/C1-AA60/3/C6). Subfraction AA60/3/C5 (322 mg) was further separated by size-exclusion chromatography (Sephadex LH-20) with MeOH gaining seven subfractions (AA60/3/C5/S1-AA60/3/C5/S7). Compound **8** (34 mg) was obtained in subfraction AA60/3/C5/S7.

### Cell lines

Human colonic adenocarcinoma cell lines (Colo 205 doxorubicin-sensitive and Colo 320/MDR-LRP multidrug-resistant expressing ABCB1 (MDR1)-LRP), ATCC-CCL-220.1 (Colo 320) and CCL-222 (Colo 205) were purchased from LGC Promochem, Teddington, UK. The cells were cultured in RPMI 1640 medium supplemented with 10% heat-inactivated foetal bovine serum, 2 mM l-glutamine, 1 mM Na-pyruvate and 100 mM Hepes. The cell lines were incubated at 37 °C, in a 5% CO_2_, 95% air atmosphere. The semi-adherent human colon cancer cells were detached with Trypsin-Versene (EDTA) solution for 5 min at 37 °C.

MRC-5 human embryonal lung fibroblast cell lines (ATCC CCL-171) were purchased from LGC Promochem, Teddington, UK. The cell line was cultured in Eagle’s Minimal Essential Medium (EMEM, containing 4.5 g/L glucose) supplemented with a non-essential amino acid mixture, a selection of vitamins and 10% heat-inactivated foetal bovine serum. The cell lines were incubated at 37 °C, in a 5% CO_2_, 95% air atmosphere.

### Bacterial strains and determination of MIC values

As Gram‐positive strains, *Staphylococcus aureus* American Type Culture Collection (ATCC) 25923 strain was used as methicillin‐susceptible reference strain; the clinical isolate *S. aureus* MRSA 272123 and *Enterococcus faecalis* ATCC 29212 strains were investigated in the study.

As Gram-negative strains *Escherichia* coli ATCC 25922 and *E. coli* AG100; *Klebsiella pneumoniae* ATCC 49619 and *Pseudomonas aeruginosa* ATCC 33362 strains were used. The MICs of compounds were determined, according to the Clinical and Laboratory Standard Institute (CLSI) guidelines (Clinical and Laboratory Standards Institute 2022).

### Assay for antiproliferative and cytotoxic effects

MRC-5 non-cancerous human embryonic lung fibroblast and human colonic adenocarcinoma cell lines (doxorubicin-sensitive Colo 205 and multidrug-resistant Colo 320 colonic adenocarcinoma cells) were used to determine the effect of compounds on cell proliferation and growth. The effects of increasing concentrations of compounds on cell proliferation and growth were tested in 96-well flat-bottomed microtitre plates. The compounds were diluted in a volume of 100 μL of medium.

The adherent human embryonal lung fibroblast cells were seeded overnight and cultured in 96-well flat-bottomed microtitre plates prior to the assay, using EMEM supplemented with 10% heat-inactivated foetal bovine serum. The density of the cells was adjusted to 6 × 10^3^ cells (antiproliferative assay) or 1 × 10^4^ cells (cytotoxicity assay) in 100 μL per well, the cells were seeded for 24 h at 37 °C, 5% CO_2_, then the medium was removed from the plates containing the cells, and the dilutions of compounds (200 µL) previously made in a separate plate were added to the cells in.

In case of the colonic adenocarcinoma cells, the 2-fold serial dilutions of compounds were prepared in 100 μL of RPMI 1640, horizontally. The semi-adherent colonic adenocarcinoma cells were treated with Trypsin-Versene (EDTA) solution. The density of the cells was adjusted to 6 × 10^3^ cells (antiproliferative assay) or 1 × 10^4^ cells (cytotoxicity assay) in 100 μL of RPMI 1640 medium, and was added to each well, with the exception of the medium control wells. The final volume of the wells containing compounds and cells was 200 μL.

The culture plates were incubated at 37 °C for 72 h (antiproliferative test) or 24 h (cytotoxicity test); at the end of the incubation period, 20 μL of MTT (thiazolyl blue tetrazolium bromide, Sigma, St. Louis, MO, USA) solution (from a stock solution of 5 mg/mL) were added to each well. After incubation at 37 °C for 4 h, 100 μL of sodium dodecyl sulphate (SDS) (Sigma) solution (10% in 0.01 M HCI) were added to each well and the plates were further incubated at 37 °C overnight. Cell growth was determined by measuring the optical density (OD) at 540/630 nm with Multiscan EX ELISA reader (Thermo Labsystems, Cheshire, WA, USA). Inhibition of the cell growth was determined according to the formula below:

$$\text{Inhibition \%}=100-[\frac{\text{OD sample}-\text{OD medium control}}{\text{OD cell control}-\text{OD medium control}}]\times 100$$

Results are expressed in terms of IC_50_, defined as the inhibitory dose that reduces the growth of the cells exposed to the tested compounds by 50%.

### Evaluation of rhodamine 123 (R123) retention by flow cytometry (inhibition of P-glycoprotein or ABCB1)

This method is a fluorescence-based detection system which uses tariquidar as a reference inhibitor of the ABCB1 efflux pump (EP). The colonic adenocarcinoma cells (the doxorubicin-sensitive Colo 205 and the multidrug-resistant Colo 320) were adjusted to a density of 2 × 10^6^/mL, re-suspended in serum-free RPMI 1640 medium and distributed in 0.5 mL aliquots into Eppendorf centrifuge tubes. The compounds were added at different concentrations (2 μM and 20 μM final concentrations, respectively), and the samples were incubated for 10 min at room temperature. Next, 10 μL (5.2 μM final concentration) of rhodamine 123 was added to the samples and the cells were incubated for 20 minutes at 37 °C, washed twice with phosphate-buffered saline (PBS) and re-suspended in 1 mL PBS for analysis. The fluorescence intensity of the cell population was measured with a Partec CyFlow flow cytometer (Partec, Munster, Germany). Tariquidar was used as a positive control at 0.2 μM final concentration in the rhodamine 123 exclusion experiments. The mean fluorescence intensity (%) was calculated for the treated MDR Colo 320 and sensitive Colo 205 cell lines as compared to the untreated cells. The fluorescence activity ratio (FAR) was calculated based on the following equation which relates the measured fluorescence values:

$$\text{FAR}=\frac{{\text{Colo}\;320}_{\text{treated}}/{\text{Colo}\;320}_{\text{control}}}{{\text{Colo}\;205}_{\text{treated}}/{\text{Colo}\;205}_{\text{control}}}$$

### Checkerboard combination assay

A checkerboard microplate method was applied to study the effect of drug interactions between the compounds and doxorubicin. The assay was carried out using multidrug-resistant Colo 320 colonic adenocarcinoma cells expressing the ABCB1 transporter. The final concentration of the chemotherapeutic agent doxorubicin used in the combination experiment was chosen in accordance with their antiproliferative activity previously determined. The dilutions of the chemotherapeutic drug were made in a horizontal direction in 100 μL, and the dilutions of the compounds vertically in the microtitre plate in 50 μL volume. The cells were re-suspended in culture medium and distributed into each well in 50 μL containing 6 × 10^3^ cells each. The plates were incubated for 72 h at 37 °C in 5% CO_2_ atmosphere. The cell growth rate was determined after MTT staining. At the end of the incubation period, 20 μL of MTT (thiazolyl blue tetrazolium bromide, Sigma) solution (from a stock solution of 5 mg/mL) was added to each well. After incubation at 37 °C for 4 h, 100 μL of SDS (Sigma-Aldrich, St. Louis, MO, USA) solution (10% in 0.01 M HCl; Merck, Darmstadt, Germany) were added to each well and the plates were further incubated at 37 °C overnight. OD was measured at 540/630 nm with Multiscan EX ELISA reader (Thermo Labsystems, Cheshire, WA). Combination index (CI) values at 50% of the growth inhibition dose (ED_50_), were determined using CompuSyn software (ComboSyn, Inc., Paramus, NJ) to plot four to five data points to each ratio. CI values were calculated by means of the median-effect equation, according to the Chou-Talalay method, where CI <1, CI = 1, and CI >1 represent synergism, additive effect (or no interaction) and antagonism, respectively.

### Real-time ethidium bromide accumulation assay

The minimum inhibitory concentrations (MICs) of the substances were determined as a prelude to the experiment. The substances did not have a significant effect on any of the bacterial strains. The MIC value was >100 µM in all cases. The bacteria were incubated in Mueller–Hinton (MH) broth.

The impact of compounds on EB accumulation was determined by the automated EB method using a CLARIOstar Plus plate reader (BMG Labtech, Aylesbury, UK). First, the bacterial strain was incubated until it reached an OD of 0.6 at 600 nm [*Escherichia coli* AG100 is incubated in Luria–Bertani (LB) broth and *Staphylococcus aureus* ATCC 25923 is incubated in tryptic soy broth (TSB)]. The culture was washed with PBS (pH 7.4) and centrifuged at 13,000 × *g* for 3 min, the cell pellet was re-suspended in PBS. The compounds were added at ½ MIC concentration (in case of >100 the concentration is 100 µM) to PBS containing a non-toxic concentration of EB (2 µg/mL). Then, 50 μL of the EB solution containing the compound were transferred into 96-well black microtitre plate (Greiner Bio-One Hungary Kft, Hungary), and 50 μL of bacterial suspension (OD_600_ 0.6) were added to the each well. Then, the plates were placed into the CLARIOstar plate reader, and the fluorescence was monitored at excitation and emission wavelengths of 525 and 615 nm every minute for 1 h on a real-time basis. From the real-time data, the activity of the compounds, namely the relative fluorescence index (RFI) of the last time point (60 min) of the EB accumulation assay, was calculated according to the following formula:

$$\mathrm{RFI}=\frac{\left(\mathrm{RFtreated}\;–\;\mathrm{RFuntreated}\right)}{\mathrm{RFuntreated}}$$

where RFtreated is the relative fluorescence (RF) at the last time point of EB retention curve in the presence of an inhibitor, and RFuntreated is the RF at the last time point of the EB retention curve of the untreated control having the solvent control (DMSO). (We also had a negative control: bacteria and EB solution only.)

Positive controls: E. coli AG100 → CCCP (50 μM)

S. aureus ATCC 25923 → reserpine (25 μM)

## Results

### Structure determination of the isolated compounds

**Figure 1.**
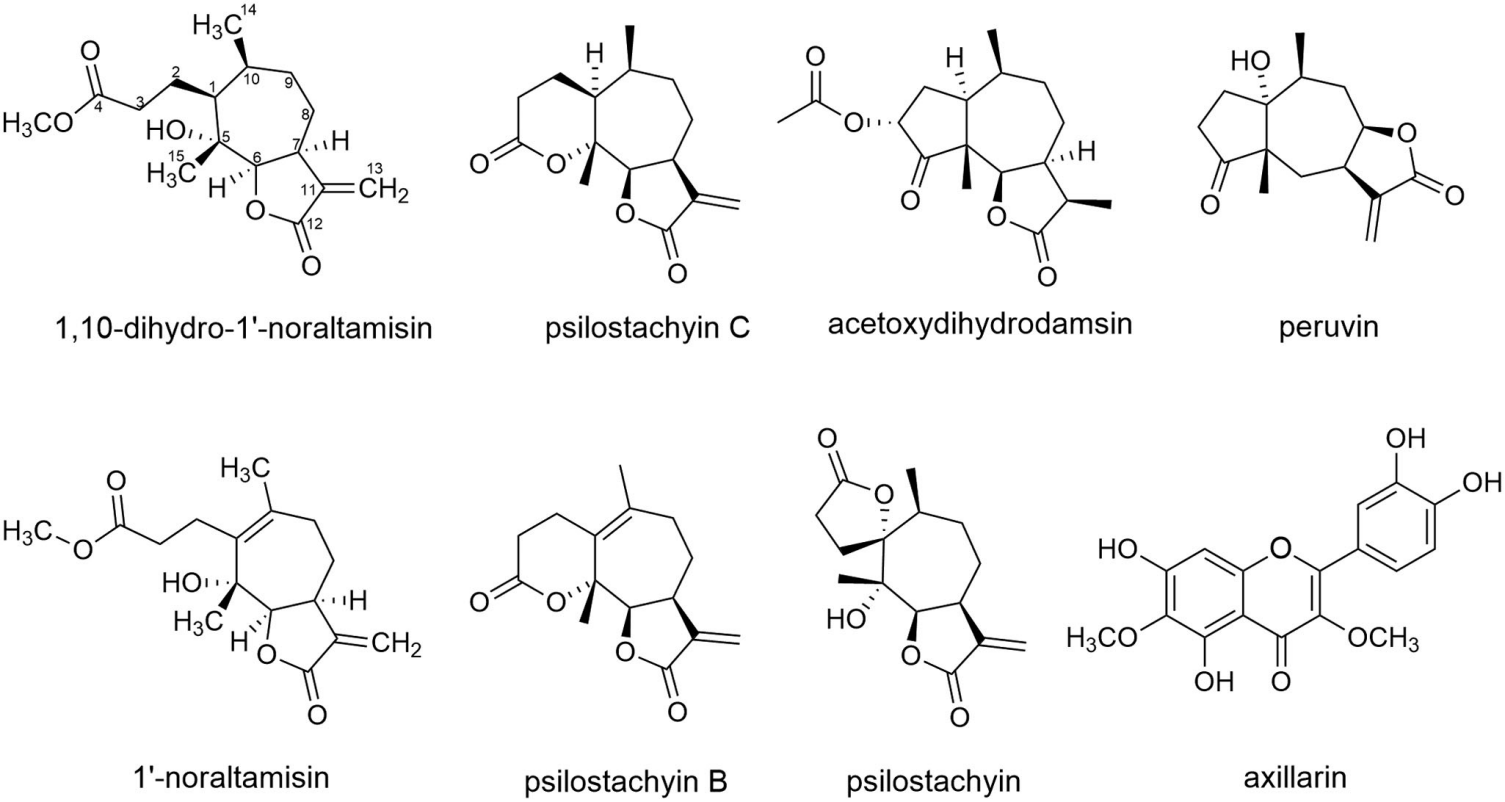
The structure of the isolated compounds from *Ambrosia artemisiifolia.*

The structure elucidation process was carried out by 1D (^1^H, ^13^C JMOD) and 2D (HSQC, HMBC, ^1^H-^1^H COSY and NOESY) NMR experiments. By comparison of the spectroscopic data with the literature data, the seven known isolated compounds were identified as psilostachyin C (**1**) (Kagan et al. 1966), acetoxydihydrodamsin (**2**) (Taglialatela-Scafati et al. 2012), peruvin (**3**) (Oberti et al. 1986), psilostachyin (**4**) (Borges-del-Castillo et al. 1981), 1′ -noraltamisin (**5**) (Delgado et al. 1988), psilostachyin B (**6**) (Oberti et al. 1986) and axillarin (**8**) (Ahmad et al. 2006) (Figure 1).

**Figure 2.**
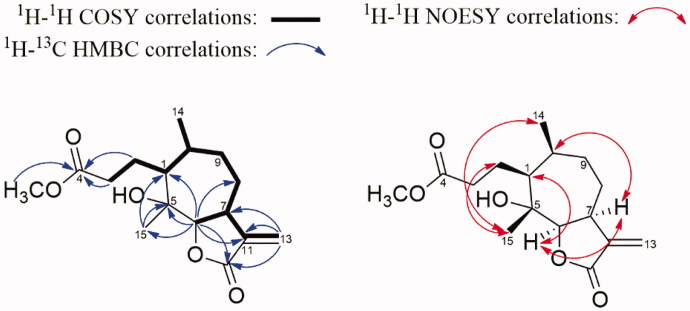
The 1H-1H COSY, key HMBC and key NOESY correlations of 1,10-dihydro-1'-noraltamisin (**7**).

Compound **7** was isolated as a white amorphous powder. The molecular formula was identified as C_16_H_24_O_5_ by HR-ESIMS at *m/z* 297.16947 [M + H]^+^ (calcd for C_16_H_25_O_5_ 297.16965), indicating 5 unsaturated degrees. The ^1^H-NMR spectrum of **7** revealed two methyl groups, one methoxy, one unsaturated methylene, and one oxygenated methine (Table 1). The ^13^C-JMOD spectrum, together with the assistant of HSQC and HMBC spectra of **7**, indicated 16 carbon signals, including three methyls, one olefinic methylene, four *sp*^3^ methylenes, four *sp*^3^ methines, one olefinic quaternary carbon, one oxygenated quaternary carbon and two carbonyl carbons (Table 1). The ^1^H-^1^H COSY together with HSQC spectra of **7** clearly revealed the COSY correlations of CH-1 (*δ*_H_ 1.59; *δ*_C_ 53.0)/CH_2_-2 (*δ*_H_ 1.92, 1.46; *δ*_C_ 21.8)/CH_2_-3 (*δ*_H_ 2.35, 2H; *δ*_C_ 36.4), CH-6 (*δ*_H_ 4.55; *δ*_C_ 89.6)/CH-7 (*δ*_H_ 3.49; *δ*_C_ 41.9)/CH_2_-8 (*δ*_H_ 2.23, 1.86; *δ*_C_ 36.3)/CH_2_-9 (*δ*_H_ 1.59, 1.46; *δ*_C_ 29.6)/CH-10 (*δ*_H_ 2.32; *δ*_C_ 34.0)/CH-1, CH-10/CH_3_-14 (*δ*_H_ 0.94, 3H; *δ*_C_ 19.1), and an allylic four-bond coupling between H-7 and the olefinic methylene CH_2_-13 (*δ*_H_ 6.23, 5.59; *δ*_C_ 121.7) (Figure 2). Furthermore, the HMBC correlations from H_2_-2, H_2_-3, and a methoxy group (*δ*_H_ 3.65, 3H; *δ*_C_ 52.0) to the carbonyl carbon C-4 (*δ*_C_ 175.9), respectively, indicated a methyl propionate moiety located at C-1. In addition, the HMBC correlations from H-6 to C-1, the oxygenated quaternary carbon C-5 (*δ*_C_ 77.1) and the methyl CH_3_-15 (*δ*_H_ 1.27, 3H; *δ*_C_ 20.6), as well as from H_3_-15 to C-1 and C-5, suggested the presence of a 1,3-dimethylcycloheptan-1-ol structure in **7**. Moreover, the HMBC correlations from H-6 to the olefinic quaternary carbon C-11 (*δ*_C_ 141.0) and another carbonyl carbon C-12 (*δ*_C_ 172.6), and from H_2_-13 to C-7, C-11 and C-12, indicated an α-methylene-γ-butyrolactone system connected at C-6 and C-7 in existence (Figure 2). Based on the above analysis, compound **7** can be identified as a rare *seco*-psilostachyinolide (Borges-del-Castillo et al. 1981; Delgado et al. 1988). Additionally, comparing the NMR data of **7** with those of psilostachyin C hydroxyethyl ester derivative (Kagan et al. 1966) and similar structures (Oberti et al. 1986; Delgado et al. 1988) suggested that the planar structure of **7** was the same as the psilostachyin C derivative. However, the psilostachyin C derivative was only reported as a synthetic product without any structural elucidation and was never named in the literature (Kagan et al. 1966). In this work, we successfully obtained compound **7** from natural sources and completely revealed its NMR spectra data and structural analysis for the first time. Further, the NOESY cross-peaks between H-1/H-6/H-7/H-10 and H-2 (*δ*_H_ 1.92)/H_3_-15/H_3_-14, as well as comparing with psilostachyin C (Kagan et al. 1966), indicated the relative configuration of **7** as shown on Figure 1 and named as 1,10-dihydro-1′-noraltamisin.

**Table 1.** The ^1^H and ^13^C-NMR data of 1,10-dihydro-1'-noraltamisin (**7**).

C/H	*δ*_H_,^a^ mult. (*J* in Hz)	*δ*_C_,^b^ type
**1**	1.59, m	53.0, CH
**2**	1.46, m; 1.92, m	21.8, CH_2_
**3**	2.35, m (2H)	36.4, CH_2_
**4**	–	175.9, C
**5**	–	77.1, C
**6**	4.55, d (9.5)	89.6, CH
**7**	3.49, m	41.9, CH
**8**	1.86, m; 2.23, m	36.3, CH_2_
**9**	1.46, m; 1.59, m	29.6, CH_2_
**10**	2.32, m	34.0, CH
**11**	–	141.0, C
**12**	–	172.6, C
**13**	5.59, d (3.5); 6.23, d (3.5)	121.7, CH_2_
**14**	0.94, d (7.0, 3H)	19.1, CH_3_
**15**	1.27, s (3H)	20.6, CH_3_
OCH_3_	3.65, s (3H)	52.0, CH_3_

^a^500 MHz, CD_3_OD; ^b^125 MHz, CD_3_OD.

### Antibacterial effects of compounds

None of the tested compounds exerted antibacterial effect. The MIC values on Gram-negative and -positive bacteria were above 100 µM.

### Antiproliferative and cytotoxic assays

To assess the *in vitro* antiproliferative and cytotoxic activity of the isolated compounds a MTT assay was performed using two human adenocarcinoma cell lines (the doxorubicin-sensitive Colo 205 and the multidrug-resistant Colo 320 colonic adenocarcinoma cell lines). Compound **2** exhibited cytotoxic activity (IC_50_ value of 7.64 ± 0.37 µM) on sensitive (Colo 205) cell line after 24 h of incubation (Table 2). Among the *seco*-psilostachyinolides, **1** showed the strongest cytotoxic effects with IC_50_ values of 26.6 ± 0.48 µM on the doxorubicin-sensitive Colo 205 cell line, while compound **4** had cytotoxic effects on the multidrug-resistant Colo 320 cell line (IC_50_ value 17.7 ± 0.20 µM). In contrast, compound **6** which shares the same *seco* derivative skeleton had no cytotoxic effects with the tested cell lines (Table 2). Besides the isolated sesquiterpene lactones, the quercetagetin 3,6-dimethyl ether derivative (**8**) showed no cytotoxic effects on the tested cell lines after a short time exposure (Table 2).

**Table 2.** Cytotoxicity of compounds **1–8** isolated from *A. artemisiifolia* (IC_50_ values µM).

Compounds	Cell lines		
	MRC-5	Colo205	Colo320
**1**	52.69 ± 2.62	26.60 ± 0.48	28.71 ± 0.11
**2**	23.77 ± 1.06	7.64 ± 0.37	10.75 ± 0.22
**3**	11.89 ± 1.45	64.44 ± 1.78	82.37 ± 1.17
**4**	41.82 ± 2.43	42.43 ± 2.37	40.08 ± 1.31
**5**	>100	>100	>100
**6**	37.57 ± 2.51	24.50 ± 0.45	17.7 ± 0.20
**7**	>100	53.57 ± 0.87	86.59 ± 1.06
8	>100	>100	>100

After a 72 h incubation period, compound **2** had the strongest antiproliferative activity among the isolated sesquiterpenes on both adenocarcinoma cell lines (IC_50_ value of 5.14 ± 0.55 µM on Colo 205 and IC_50_ value of 3.67 ± 0.35 µM on Colo 320, Table 3). Compound **6** was inactive in this assay as well, however, compound **8** inhibited the cell proliferation of the human embryonal lung fibroblast cells at very low concentration (IC_50_ value of 4.03 ± 0.56 µM) (Table 3).

**Table 3.** Antiproliferative effects of compounds **1–8** isolated from *A. artemisiifolia* and of the positive control doxorubicin (IC_50_ values in µM).

Compounds	Cell lines		
	MRC-5	Colo205	Colo320
**1**	35.13 ± 4.03	15.61 ± 0.83	14.66 ± 0.82
**2**	10.96 ± 0.31	5.14 ± 0.55	3.67 ± 0.35
**3**	26.42 ± 1.30	26.35 ± 1.12	21.19 ± 0.72
**4**	26.72 ± 0.51	14.37 ± 1.00	8.78 ± 0.22
**5**	>100	>100	>100
**6**	26.36 ± 0.81	10.99 ± 0.56	5.29 ± 0.15
**7**	39.78 ± 0.53	17.01 ± 1.99	34.51 ± 2.07
**8**	4.03 ± 0.56	66.75 ± 0.96	50.40 ± 2.98
Doxorubicin	0.53 ± 0.06	0.24 ± 0.03	0.14 ± 0.03

Compound **2** exerted selective cytotoxicity towards the human colonic adenocarcinoma cell lines in comparison with its activity against human embryonal lung fibroblast cell line (selectivity index [SI] = 3.11). In contrast, compounds **4** and **6** showed cell proliferation inhibitory activities on the Colo320 multidrug-resistant cell line with IC_50_ values of 8.78 ± 0.22 µM, 5.29 ± 0.15 µM, and the molecules had relatively moderate selectivity against the MRC-5 cell line (SI = 3.04 and SI = 4.98).

### Checkboard combination assay

The drug interactions between the compounds and doxorubicin were assessed using multidrug-resistant Colo320 colonic adenocarcinoma cells which expressing the ABCB1 transporter. Compounds **7** and **8** have synergistic effects with the doxorubicin at certain concentrations. However, compounds **1**–**3** showed antagonistic effects at certain concentrations (Table 4).

**Table 4.** The drug interactions between the compounds and doxorubicin.

Compounds	Starting concentration	Ratio^a^	CI at ED_50_^b^	SD	Type of interaction
**1**	50 µM	5.8:1	3.71	0.73	Strong antagonism
		11.6:1	3.34	0.43	Strong antagonism
		23.2:1	2.55	0.31	Antagonism
		46.4:1	1.13	0.07	Slight antagonism
		92.8:1	1.47	0.28	Antagonism
		185:1	1.34	0.28	Moderate antagonism
**2**	15 µM	1.74:1	2.45	0.48	Antagonism
		3.48:1	2.37	0.16	Antagonism
		6.96:1	1.48	0.22	Antagonism
		13.92.1	1.55	0.15	Antagonism
		27.84:1	1.22	0.23	Moderate antagonism
		55.68:1	1.34	0.29	Moderate antagonism
**3**	65 µM	7.54:1	1.28	0.16	Moderate antagonism
		15.08:1	1.7	0.23	Antagonism
		30.16:1	2.34	0.3	Antagonism
		60.32.1	1.4	0.024	Moderate antagonism
		120.64:1	1.7	0.11	Antagonism
		241.28:1	1.01	0.18	Additive effect
**4**	25 µM	2.9:1	0.95	0.06	Additive effect
		5.8:1	1.44	0.32	Moderate antagonism
		11.6:1	0.67	0.14	Synergism
		23.2:1	2.26	0.42	Antagonism
		46.4:1	1.39	0.07	Moderate antagonism
		92.2:1	1.16	0.21	Slight antagonism
**5**	30 µM	3.48:1	2.62	0.62	Antagonism
		6.69:1	1.02	0.072	Additive effect
		13.92:1	0.88	0.043	Slight synergism
		27.84:1	1.09	0.039	Additive effect
		55.68:1	1.33	0.036	Moderate antagonism
		111.36:1	0.94	0.28	Additive effect
**6**	100 µM	11.6:1	0.4	0.14	Syn.
		23.2:1	0.34	0.06	Syn.
		46.4:1	2.6	0.36	Antagonism
		92.8:1	1.13	0.09	Slight antagonism
		185.6:1	1.4	0.2	Moderate ant.
		371.2:1	1.5	0.16	Antagonism
**7**	150 µM	17.42:1	0.1	0.03	Very strong syn.
		34.84:1	0.79	0.02	Moderate syn.
		69.68:1	0.74	0.03	Moderate syn.
		139.36:1	0.74	0.05	Moderate syn.
		278.72:1	0.75	0.7	Moderate syn.
		557.44:1	0.51	0.05	Syn.

^a^Ratio: the best combination ratio between compounds and doxorubicin.

^b^CI at ED_50_: combination index value at the 50% growth inhibition dose.

Combination index (CI): 0–0.1: very strong synergism, 0.1–0.3: strong synergism, 0.3–0.7: synergism, 0.7–0.85: moderate synergism, 0.85–0.9: slight synergism, 0.9–1.1: additive effect, 1.1–1.2: slight antagonism, 1.2–1.45: moderate antagonism, 1.45–3.3: antagonism, 3.3–10: strong antagonism and >10: very strong antagonism.

### ABCB1 efflux pump (P-glycoprotein) inhibitory assay

In this study, we used tariquidar (FAR value: 6.6 at 0.2 µM) as a reference inhibitor of the P-glycoprotein EP expressed by the two adenocarcinoma cell lines. The fluorescence dye, rhodamine 123 was used as a marker of the ABCB1 transporter activity to explore the potential inhibitory activity of the isolated compounds. Inside cells without overexpressing the P-glycoprotein, the rhodamine 123 accumulates and resulting in an increase in fluorescence intensity (*λ*_ex_ = 490/*λ*_em_ = 525 nm). The multidrug resistance (MDR) cells overexpressing the EP can easily remove the dye which results the rapid decreasing the intensity of cellular fluorescence. If the fluorescence activity ratio (FAR) value is above 2, it is conventionally accepted that the subjected compound can be a good ABCB1 inhibitor. After measuring the fluorescence intensity of the cell population none of the compounds showed adequate inhibitory effects according to their FAR values. The FAR values of the tested compounds were in the range of 0.2-1-1.

### The ethidium bromide (EB) accumulation assay

The EB accumulation assay gives insight information about the function of the EP expressed by *Escherichia coli* AG100 and *Staphylococcus aureus* ATCC 25923. The effective EP inhibitors increase the level of fluorescence of the intercalating agent EB (used as fluorescence dye) because of its accumulation within the bacteria. The EP inhibiting activity of the isolated compounds was evaluated based on the RFI of the real-time accumulation curves. In this study, none of the sesquiterpenes showed EP inhibitory activity. The RFI values were around 0, that is, equal to the untreated sample. RFI values obtained for positive controls are as follows: reserpine (RFI = 2.77) on *S. aureus* ATCC 25923 and CCCP (carbonyl cyanide *m*-chlorophenylhydrazine) (RFI = 1.34) on *E. coli* AG100.

## Discussion

Eight compounds were isolated from the MeOH extract of the aerial parts of *A. artemisiifolia* and based on HR-MS and NMR experiments their structures were identified as the sesquiterpenes psilostachyin C (**1**), acetoxydihydrodamsin (**2**), peruvin (**3**), psilostachyin (**4**), 1′-noraltamisin (**5**), psilostachyin B (**6**), 1,10-dihydro-1′-noraltamisin (**7**) and the flavonoid axillarin (**8**). After comparing NMR data of 1,10-dihydro-1′-noraltamisin (**7**) with those found in the literature, it was realized that this compound was obtained for the first time from the plant. It belongs to a rare class of *seco*-psilostachyinolides bearing an open ring system. It can be hypothesized that the biosynthesis of this class of *seco* derivatives takes place in two independent pathways: the mevalonic acid (MVA) pathway in the cytoplasm or the 2-C-methyl-d-erythritol-4-phosphate (MEP) pathway in the plastids (Bick and Lange 2003). The precursor in both cases, 2*e*,6*e*-farnesyl pyrophosphate (FPP), results from the addition of a molecule of IPP onto GPP. The skeleta of this open ring system arise from the cyclodecadiene-type product of the cyclization of FPP, or of its geometrical isomer at C-2 (2*z*,6*e*-FPP), or of nerolidyl pyrophosphate, by nucleophilic attack on the distal double bond which leads to the germacradienyl cation, from which may arise also the other lactone skeletal types (Jean 1999). Until now just a few of this type of seco derivatives were reported in the literature from the genus *Ambrosia*. 1′-Noraltamisin (**5**) was reported only from a Mexican population (collected in North Zacatecas) of *Ambrosia confertiflora* DC (Delgado et al. 1988). Structurally similar sesquiterpene lactones, altamisin from *A. peruviana* (Central American collection) (Borges et al. 1978) and altamisic acid from *A. tenuifolia* (collection form North Central Argentina), were also reported (Oberti et al. 1986). From a Yugoslavian collection of *A. artemisiifolia,* Stefanović et al (1987) isolated 4-oxo-3,4-*seco*-ambrosan-6,12-olide-3-oic acid; the same compound was also found by Taglialatela-Scafati et al. (2012) from an Italian plant sample.

The cytotoxic and antiproliferative activities of the sesquiterpene lactones are the most characteristic bioactivities in this naturally occurring secondary metabolites. To assess these activities, we used two different experimental setups of MTT assay. This allows the comparison of results obtained from a short-term (24 h) treatment of a relatively higher number of cells (cytotoxic activity) and those from a long-term (72 h) treatment of a lower cell number (cytostatic or antiproliferative activity). In the *in vitro* studies, acetoxydihydrodamsin (**2**) showed the most potent cytotoxic effect on sensitive (Colo 205) cell line. After a long-term exposure to the above-mentioned compound, a strong antiproliferative effect was also observed on Colo205 and Colo320 adenocarcinoma cell lines. The *seco*-psilostachyinolides, 1′-noraltamisin (**5**) and psilostachyin (**4**) have significant antiproliferative effects on the multidrug-resistant Colo 320 cell line and showed moderate selectivity against human embryonal lung fibroblast cell line. It is generally accepted that the presence of a C-11/C-13 exocyclic double bound conjugated to the γ-lactone is crucial for cytotoxicity of this type of compounds and as noted by several authors due to this moiety these secondary metabolites can alkylate the nucleophilic groups of enzymes (Kreuger et al. 2012). In spite all of these, in the case of acetoxydihydrodamsin (**2**), the lack of this moiety is conspicuous, compared to the other sesquiterpenes. Instead of this, compound **2** bears an acetyl group at C-3, which demonstrates that the presence of an α,β-unsaturated carbonyl group is not required for the inhibition of cell proliferation. In addition to the isolated sesquiterpene lactones, the quercetagetin 3,6-dimethyl ether derivative axillarin (**8**) showed antiproliferative effects on MRC-5 cells with surprisingly low concentration and no selectivity towards to this normal cell line, compared to the sesquiterpenes.

Cancer cells can develop MDR against to a broad spectrum of chemotherapeutic agents which results failure in the treatment of cancer and a major challenge in the drug development to overcome this problem. The MDR cells overexpressing the ATP binding ABC transmembrane proteins can easily efflux various chemically diverse or potentially dangerous compounds across the cell membrane, e.g., anticancer drugs, which leads to chemoresistance (Gottesman and Ambudkar 2001; Dean and Annilo 2005). The ABCB1 EP the member of this transmembrane proteins can reduce the cellular uptake of drugs into cancer cells and protect them from medical interventions (Doyle et al. 1998). Naturally occurring substances which may inhibit this type of ABC transporters have the potential to amplify the efficacy of anticancer drug. In the P-glycoprotein (ABCB1) inhibitory assay none of the tested sesquiterpenes, nor the flavonoid derivative have considerable effects on the ABCB1 EP function. These findings suggest that this type of plant secondary metabolite expresses their inhibitory activity inside the cancer cells via other mechanisms, e.g., inhibition of DNA methylation (Liu et al. 2009), inhibition of the NF-κB signalling pathway (Kreuger et al. 2012), cell cycle checkpoint inhibition (Sturgeon et al. 2005), inducing apoptosis and cell cycle arrest (Martino et al. 2015).

After the EB accumulation assay, we concluded that the isolated *Ambrosia* sesquiterpenes could not be considered as effective bacterial EP inhibitors.

Even though the investigated Asteraceae species is an invasive and highly allergenic weed, the plant’s secondary metabolites dispose noteworthy cytotoxic and antiproliferative activity on the investigated adenocarcinoma cell lines. However, further study is still necessary to elucidate the structure–activity relationships and the molecular mechanisms of these compounds. Also, chemical modifications and semisynthetic derivatives may lead more effective and selective substances towards cancer cells.
